# Intestinal Cell Tight Junctions Limit Invasion of *Candida albicans* through Active Penetration and Endocytosis in the Early Stages of the Interaction of the Fungus with the Intestinal Barrier

**DOI:** 10.1371/journal.pone.0149159

**Published:** 2016-03-02

**Authors:** Marianne Goyer, Alicia Loiselet, Fabienne Bon, Coralie L’Ollivier, Michael Laue, Gudrun Holland, Alain Bonnin, Frederic Dalle

**Affiliations:** 1 UMR 1347, Univ Bourgogne-Franche Comté, 17 Rue Sully, BP 86 510, F-21065 Dijon Cedex, France; 2 Centre Hospitalier Universitaire, Service de Parasitologie Mycologie, 2 Rue Angélique Ducoudray, F-21079 Dijon Cedex, France; 3 Laboratoire de Parasitologie-Mycologie, Aix-Marseille Univ. Marseille; AP-HM, CHU Timone, F-13385 Marseille cedex 05, France; 4 Robert Koch-Institute, Centre for Biological Threats and Special Pathogens, Advanced Light and Electron Microscopy, Nordufer 20, 13353 Berlin, Germany; David Geffen School of Medicine at University of California Los Angeles, UNITED STATES

## Abstract

*C*. *albicans* is a commensal yeast of the mucous membranes in healthy humans that can also cause disseminated candidiasis, mainly originating from the digestive tract, in vulnerable patients. It is necessary to understand the cellular and molecular mechanisms of the interaction of *C*. *albicans* with enterocytes to better understand the basis of commensalism and pathogenicity of the yeast and to improve the management of disseminated candidiasis. In this study, we investigated the kinetics of tight junction (TJ) formation in parallel with the invasion of *C*. *albicans* into the Caco-2 intestinal cell line. Using invasiveness assays on Caco-2 cells displaying pharmacologically altered TJ (*i*.*e*. differentiated epithelial cells treated with EGTA or patulin), we were able to demonstrate that TJ protect enterocytes against invasion of *C*. *albicans*. Moreover, treatment with a pharmacological inhibitor of endocytosis decreased invasion of the fungus into Caco-2 cells displaying altered TJ, suggesting that facilitating access of the yeast to the basolateral side of intestinal cells promotes endocytosis of *C*. *albicans* in its hyphal form. These data were supported by SEM observations of differentiated Caco-2 cells displaying altered TJ, which highlighted membrane protrusions engulfing *C*. *albicans* hyphae. We furthermore demonstrated that Als3, a hypha-specific *C*. *albicans* invasin, facilitates internalization of the fungus by active penetration and induced endocytosis by differentiated Caco-2 cells displaying altered TJ. However, our observations failed to demonstrate binding of Als3 to E-cadherin as the trigger mechanism of endocytosis of *C*. *albicans* into differentiated Caco-2 cells displaying altered TJ.

## Introduction

*Candida albicans* is a dimorphic yeast and is the most frequently isolated yeast in humans. It belongs to the natural microbial flora and resides as a commensal at the mucosal surfaces (*i*.*e*. the digestive tract, vagina or oral cavity) of healthy individuals [1, 2]. This commensalism results from a subtle balance between (*i*) the host’s immune defences, the bacterial flora and other local environmental conditions and (*ii*) the yeast’s virulence factors. However, disturbance of this balance can lead to pathogenicity of the yeast, which can proliferate and colonize the different mucosal surfaces before invading the tissues [3]. As a consequence, this normally harmless microbial agent can cause superficial to severe systemic infections mostly originating from the gastrointestinal tract [4, 5]. Indeed, *C*. *albicans* cells invade through the epithelial barrier of the gut into the bloodstream and spread to other organs [6]. *C*. *albicans* is the fourth most common infectious agent isolated from blood cultures. It is responsible for 60% of bloodstream infections due to fungi and isolated in 8 to 15% of haematogenous nosocomial infections. Moreover, systemic infections have an attributable mortality rate of approximately 40% [7].

During mucosal colonization and invasion, *C*. *albicans* faces different types of epithelial cells, which influences the mechanisms by which the fungus invades these cells [8]. Concerning invasion into host oral epithelial cells, two different mechanisms of *C*. *albicans* have been observed: (i) active penetration of the hyphal form due to the physical forces of hyphae production associated with the release of lytic enzymes such as members of the secreted aspatyl proteinases (SAPs) family and (ii) an active mechanism of the oral epithelial cell, which is able to endocytose the hyphal form [8, 9]. Interestingly, concerning the intestine, active penetration of fungal hyphae into intestinal cells was reported whereas endocytosis of *C*. *albicans* by intestinal cells was not observed [8].

Epithelial cells of the oral cavity and respiratory, urinary, and digestive tracts form an interface between a pathogen-rich lumen and an underlying basal zone of immune cells. They are a major target for invasive micro-organisms, including fungi, as well as a site of intense host defences. Epithelial cells are armed with antimicrobial innate immune functions that prevent pathogen attachment and infection, including the release of soluble receptor fragments. Another major factor against pathogen entry is epithelial integrity, which is mediated by cell junctions [10]. However, epithelia from different anatomical sites can display structural differences, including the spatial organisation of epithelial cells and the composition of cell junctions. Indeed, the oral mucosa lining the inside of the mouth consists of a stratified multicellular layer of keratinized epithelial cells. Epithelial oral cells interact with each other through different weak junctions including Adherens and Gap junctions as well as Desmosomes. In contrast, intestinal epithelial cells (IEC) are composed of a monolayer of differentiated and polarized enterocytes which display an apical side composed of microvilli which form the brush border that is exposed to the digestive lumen. This monolayer of enterocytes ensures the integrity and impermeability of the intestinal barrier thanks to a complex hierarchical system of intercellular junctions including weak junctions (*i*.*e*. Adherens and Gap junctions, Desmosomes) and Tight Junctions (TJ), which are the most apically located intercellular junctions. Adherens Junctions (AJ) are located immediately below TJ and are mainly composed of proteins of the cadherin family including E-cadherin. AJ together with TJ form a junction belt ensuring architectural cohesion of the intestinal epithelium. Then, Desmosomes and Gap Junctions are distributed on the lateral side of the enterocyte, between AJ and the basolateral side.

Phan et *al*. reported that endocytosis of *C*. *albicans* into oral and endothelial cells was mediated by the binding of the fungal adhesin Als3 to E-cadherin or N-cadherin, respectively, [11] through an Als3’ leucin-rich domain. However, although E-cadherin is expressed by AJ at the basolateral side of enterocytes, endocytosis of *C*. *albicans* by enterocytes was not observed [8].

In this study, we hypothesized that the absence of endocytosis of *C*. *albicans* into enterocytes could result from inaccessibility of the yeast to E-cadherin at the Adherens junction site thanks to the presence of TJ sealing the apical membranes of adjacent enterocytes. We therefore investigated the endocytic potential of intestinal cells that allowed access to E-cadherin to further evaluate the role of E-cadherin in the endocytic process of *C*. *albicans* by intestinal cells. Scanning electron microscopy, fluorescence microscopy and invasiveness assays using fungal mutants and cell inhibitors were used for this study. Our data demonstrated that intestinal cells are able to internalize *C*. *albicans* through endocytosis when TJ are immature or artificially altered. However, we were not able to demonstrate the role of E-cadherin in this phenomenon.

## Experimental Procedures

### *Candida albicans* strains and growth media

*Candida albicans* SC5314 was used for all assays as a wild-type strain [12]. To analyze the role of hyphae formation in the internalization of *C*. *albicans*, the *hgc1Δ/Δ* mutant was used [13]. To analyze the role of invasin Als3 in the internalization of *C*. *albicans*, *als3Δ/Δ* mutant was used [14]. All *C*. *albicans* strains used in this study are listed in Table 1. The prototrophic strain CEC740 was constructed through transformation of strain BWP17 [15] by CIp30, an integrative plasmid harboring the *URA3*, *ARG4* and *HIS1* genes and targeting the *RPS10* locus [16], using standard transformation procedures for *C*. *albicans* [17]. All strains were maintained on solid Sabouraud dextrose agar 2%.

**Table 1:** Strains of *C*. *albicans* used in this study.

Strain	Acronym	Control strain	Genotype	Reference
**SC5314**	WT		Clinical isolate from London Mycological Reference Laboratory	[12]
**CEC740**			*ura3Δ*::*λimm434/ura3Δ*::*λimm434 arg4Δ*::*hisG/arg4Δ*::*hisG his1Δ*::*hisG/his1Δ*::*hisG RPS1/RPS1*::CIp30	[18]
**WYZ12.2**	*hgc1Δ/Δ*	CEC740	*ura3Δ*::*λimm434/ura3Δ*::*λimm434 arg4Δ*::*hisG/arg4Δ*::*hisG his1Δ*::*hisG/his1Δ*::*hisG hgc1Δ*::*HIS1/hgc1Δ*::*ARG4 RPS1/RPS1*::CIp10	[13]
**WYZ12.1**	*hgc1Δ/HGC1*	CEC740	*ura3Δ*::*λimm434/ura3Δ*::*λimm434 arg4Δ*::*hisG/arg4Δ*::*hisG his1Δ*::*hisG/his1Δ*::*hisG hgc1Δ*::*HIS1/hgc1Δ*::*ARG4*::CIp10-*HGC1*	[13]
**CAYF178U**	*als3Δ/Δ*	CEC740	*ura3Δ*::*λimm434/ura3Δ*::*λimm434*::*IRO1-URA3 arg4Δ*::*hisG/arg4Δ*::*hisG his1Δ*::*hisG/his1Δ*::*hisG als3Δ*::*HIS1/als3Δ*::*ARG4*	[14]
**CAQTP178U**	*als3Δ/ALS3*	CEC740	*ura3Δ*::*λimm434/ura3Δ*::*λimm434*::*IRO1-URA3 arg4Δ*::*hisG/arg4Δ*::*hisG his1Δ*::*hisG/his1Δ*::*hisG als3Δ*::*HIS1/als3Δ*::*ARG4*::*ALS3*	[14]

For the invasion assays, all *C*. *albicans* strains were grown as yeast forms in liquid YPD medium (yeast extract 1%, bacto-peptone 2%, and dextrose 2%) overnight at 37°C, in a shaking incubator. *C*. *albicans* cells were then diluted in fresh liquid YPD to a DO_600nm_ of 0.2 to 0.4, and then grown to log phase for another 2 h in the same conditions. *C*. *albicans* cells were then sonicated and adjusted to the desired concentration in Dulbecco’s modified Eagle’s minimum essential medium (DMEM) (Gibco, Life Technology^®^, Saint Aubin, France). For killing, hyphae of *C*. *albicans* were scraped, rinsed, re-suspended in PBS containing 0.04% Thimerosal (2-[ethylmercuriomercapto] benzoic acid sodium salt) (Sigma-Aldrich^®^, Saint-Quentin Fallavier, France) and incubated for 60 min at room temperature. After washing, *C*. *albicans* cells were counted in a hemacytometer to adjust the concentration. 100μl of Thimerosal treated yeast cells were plated on Sabouraud dextrose agar to confirm that all cells had been killed.

The absence of fungal cytotoxicity upon growth or hyphal development (length of filament after 4 h in sera at 37°C) was systematically verified during *C*. *albicans* exposure to patulin (100 μM), cytochalasin D (0.5 μM) used independently or in combination (S1 Fig).

### Cell lines and growth conditions

The cell line Caco-2 (ATCC n°HTB-37^™^) derived from a human colon adenocarcinoma was purchased from the American Type Culture Collection. Caco-2 cells display several morphological and functional characteristics of mature intestinal epithelial cells (IEC) [19] and are widely used as an *in vitro* model of the intestinal barrier.

Cells were cultured in DMEM supplemented with 10% FCS and 0.1 mM non-essential amino acids, without antibiotics or antifungal agents. Briefly, 4 to 5x10^5^ cells were seeded onto 14 mm diameter glass coverslips previously placed in 24 well plates. For TEER measurements, culture was performed on Cell Culture Inserts (polyethylene terephthalate membrane, pore size = 0.4 μm, effective growth area of membrane = 0.3 cm^2^, Falcon, BD Biosciences^®^, Le Pont de Claix, France). Cells were maintained in a humidified incubator at 37°C with 5% CO_2_ and were used for experiments 15 to 21 days after seeding, except for experiments using non-differentiated Caco-2 cells which were used 2 or 3 days after seeding (Passages 50 to 100).

### Invasion assays

Invasion assays were performed using Caco-2 cells after 2–3 days or 15–21 days post-seeding. Briefly, intestinal cells cultured on coverslips or polycarbonate inserts were infected with 10^5^ or 5x10^4^ log phase yeast cells, respectively, for 2 hours at 37°C. Invading *C*. *albicans* cells were quantified using a differential fluorescence assay as described elsewhere [8]. Briefly, the medium was discarded and coverslips were rinsed three times with PBS to remove non-adherent yeasts. The cells were then fixed with 4% PFA. The adherent part of *C*. *albicans* was stained using a rabbit polyclonal antibody to *C*. *albicans* (Acris Antibodies, Germany) (1/2000 in PBS) for 1 hour, washed in PBS and then incubated with an Alexa-Fluor 568 goat anti-rabbit antibody (Life Technology, Saint Aubin, France). Next, both adherent and internalized fungal parts were stained using a second immunofluorescence staining performed after permeabilization of the monolayers using a solution of 0.5% Triton X-100 in PBS for 10 min. Fungal cells were stained using the rabbit anti-*C*. *albicans* polyclonal antibody (Acris Antibodies, Germany) then counterstained using a second anti-rabbit antibody IgG conjugated with Alexa-Fluor 488 (Life Technology, Saint Aubin, France). Coverslips were washed with PBS, mounted and observed with a BX51 fluorescence microscope using X40 magnification (Olympus France). The percentage of invading *C*. *albicans* cells was determined by dividing the number of totally internalized cells by the total number of adherent cells. At least 100 fungal cells were counted on each coverslip, and all experiments were performed in triplicate on at least three separate occasions. The contribution of intestinal cell endocytosis in the internalization of *C*. *albicans* was investigated using cytochalasin D (CytD) and amiloride, both inhibitors of endocytosis [8, 20, 21]. Invasion assays were performed as described above except that the Caco-2 cells were pre-treated for 30 min at 37°C with amiloride 0.5mM or CytD 0.5μM (Sigma-Aldrich^®^, Saint-Quentin Fallavier, France) or with an equivalent amount of its solvent (*i*.*e*. DMSO) as a control. Caco-2 cells were further incubated with the inhibitors over the infection period. The innocuity of DMSO (*i*.*e*. the solvent used in the CytD solution), CytD 0.5μM and amiloride 0.5mM on Caco-2 cells and *Candida* cells was verified elsewhere [8].

For experiments aimed at blocking the E-cadherin protein, a mouse monoclonal anti-E cadherin antibody (SHE78-7, Invitrogen, Life Technology^®^, Saint Aubin, France) was used. SHE78-7 antibody was diluted at 2 or 4 mg/mL in fresh DMEM. Caco-2 cells were then incubated with these two different dilutions of blocking antibody for 2 hours at 37°C under 5% CO_2_ and 95% humidity. For invasion assays, the extracellular medium was then replaced with fungal or bacterial suspensions containing the E-Cadherin blocking antibody at the same concentrations as those described above.

### Modulation of Tight Junction integrity

The effect of modulating IEC TJ integrity on *C*. *albicans* invasion was investigated using the mycotoxin patulin, which has previously been described for its ability to alter specifically the integrity of the TJ [22, 23]. Differentiated Caco-2 cells were pre-incubated with patulin 100μM (Enzo, Life Sciences^®^, Villeurbanne, France) in a humidified incubator for 4 hours at 37°C with 5% CO_2_ before the invasion assays. To exclude a cytotoxic effect of patulin on IEC, Caco-2 cells were incubated with or without patulin 100μM in a humidified incubator for 4 hours at 37°C with 5% CO_2_. Monolayers of IEC were then stained with Sytox Nucleic Acid Strain ^®^ (Molecular Probes, Life Technology^®^, Saint Aubin, France) for 10 min. Immunofluorescence of the monolayers was quantified with a spectrophotometer (485/535nm) using a Victor Wallac X4 (PerkinElmer^®^, Waltham, USA). For each well, 100 measurements were performed on different parts of the IEC monolayer. The level of cytotoxicity was obtained by calculating the average OD of the 100 values previously obtained and was expressed as relative toxicity as follows: experimental OD *minus* background cells OD (*i*.*e*. IEC without pre-treatment) / mean OD of the positive controls (*i*.*e*. IEC pre-treated with 1% Triton X-100 for 10 min) included in each assays *minus* background cells OD.

Trans Epithelial Electrical Resistance (TEER) was used to monitor the integrity and the permeability of the epithelial monolayer using a Millicell-ERS volt-ohm meter (Merk Millipore^®^, Molsheim, France). Indeed, TEER reflects the impermeability of the Caco-2 cell monolayer, which correlates with the integrity of the TJ (*i*.*e*. along the differentiation of Caco-2 cells) [24, 25]. TEER values were expressed as Ω.cm^2^ after subtracting the blank filter resistance from the read measure and multiplying this result by the effective growth area of the filter insert. Because TEER values often vary among individual Caco-2 cell monolayers, the electrical resistance for each insert was recorded by performing three measurements before and after the experimental treatment, and the percentage of variation from baseline (% TEER) was calculated for each insert.

### Immunoblotting

Caco-2 cells were lysed in lysis buffer (CelLytic Mammalian Cells, Sigma Aldrich^®^, Saint-Quentin Fallavier, France) containing protease inhibitors (Complete, Roche^®^, Meylan, France). The protein concentration was measured using the BCA colorimetric method (BC Assay Protein Quantitation Small Kit, Interchim^®^, Montluçon, France) and, for each experimental condition, an equal amount of total proteins was separated on 8% SDS polyacrilamide gel and then transferred onto PVDF membranes. Blots were blocked overnight in Tris-buffered saline (TBS) containing 0.1% Tween 20 and 5% skimmed milk (Sigma Aldrich^®^, Saint-Quentin Fallavier, France). The PVDF membranes were further stained for 1 h at room temperature with a mouse monoclonal anti β-actin antibody (Ambion, Life technology^®^, Saint Aubin, France) or a mouse monoclonal anti-ZO-1 antibody (Invitrogen, Life Technology^®^, Saint Aubin, France). Blots were then incubated for 1h at room temperature with horseradish peroxidase-conjugated secondary antibodies (Dako, Agilent Technology^®^, Les Ulis, France) and revealed by chemiluminescence (ECL plus, Amersham, GE Healthcare Life Science^®^, Velizy-Villacoublay, France) before printing on radiographic film (Hyperfilm, Amersham, GE Healthcare Life Science^®^, Velizy-Villacoublay, France).

### Immunofluorescent staining of junctional proteins of IEC

Caco-2 cells grown on glass coverslips were rinsed with PBS and fixed with PFA 4%. Monolayers were next permeabilized with 0.1% Triton X-100 for 10 min. Caco-2 cells were incubated with the monoclonal mouse anti-ZO-1 antibody (Zymed, Life Technology^®^, Saint Aubin, France) or the monoclonal mouse anti-E-cadherin antibody (Zymed, Life Technology^®^, Saint Aubin, France) for 1 h at 37°C. After 3 rinses with PBS, cells were counterstained for 30 min at 37°C with a goat anti-mouse antibody, conjugated with AlexaFluor 488 (Invitrogen, Life Technology^®^, Saint Aubin, France) and AlexaFluor 568 (Invitrogen, Life Technology^®^, Saint Aubin, France) for ZO-1 and E-cadherin staining, respectively. Cellular nuclei were stained using Dapi (Invitrogen, Life Technology^®^, Saint Aubin, France) for 5min at room temperature. The coverslips were inverted, placed on glass slides and examined with a BX51 epifluorescence microscope (Olympus^®^, Tokyo, Japan).

### Quantification of Apolipoprotein A-IV gene expression by RT-qPCR

After 1, 2, 3, 5, 10 and 15 days of growth on coverslips, Caco-2 cells were lysed using PeqGold (PeqLab^®^ Biotechnology GmbH, Germany) in order to extract total mRNAs. The quality and purity of the mRNAs were checked using Agilent RNA 6000 Nano Kit and a Bioanalyser (Agilent Technologies^®^, Les Ulis, France). RT-qPCR reactions were performed using the SuperScript III Platinium SYBR Green One-Step RT-qPCR Kit (Invitrogen, Life Technology^®^, Saint Aubin, France) with ApoA-IV TaqMan Gene Expression Assays (Hs00166636_m1, Applied Biosystems, Life Technology^®^, Saint Aubin, France). Four biological replicates were assayed per time point and each was quantified in duplicate.

### Scanning electron microscopy

Caco-2 cells were cultured on polycarbonate filter inserts. Cell layers were immersed overnight in 2.5% glutaraldehyde (in 0.05 M Hepes buffer, pH 7.2) and gently washed with distilled water prior to post-fixation (1% OsO_4_, 1 h). Samples were then rinsed with distilled water, dehydrated with alcohol (stepwise 30–96%), dried (critical point dried in CO_2_ (CPD 030, BAL TEC, Vaduz, Liechtenstein)) and sputter-coated with 4 nm gold-palladium (Polaron Sputter Coating Unit E 5100, GaLa Instrumente, Bad Schwalbach). The samples were examined using a LEO 1530 Scanning Electron Microscope (Carl Zeiss SMT AG, Oberkochen) operated at 3 kV.

### Statistical analyses

R 3.1.2 software was used for all statistical analyses (http://www.r-project.org/). The parametric Welch’s t-test (or unequal variances t-test, “t.test” function) was used for the comparison of means from two independent groups after verification of assumptions (normal distribution for the populations of random errors). When assumptions were not verified, data were analyzed by the nonparametric Mann-Whitney U test (“wilcox.test” function) for the comparison of two independent groups. ANOVA (“lm” function) was used for the comparison of means of several groups, after verification of assumptions (homogeneity of variances and normal distribution for the populations of random errors).

## Results

### Differentiation of intestinal cells limits the invasiveness of *C*. *albicans*

**Fig 1:**
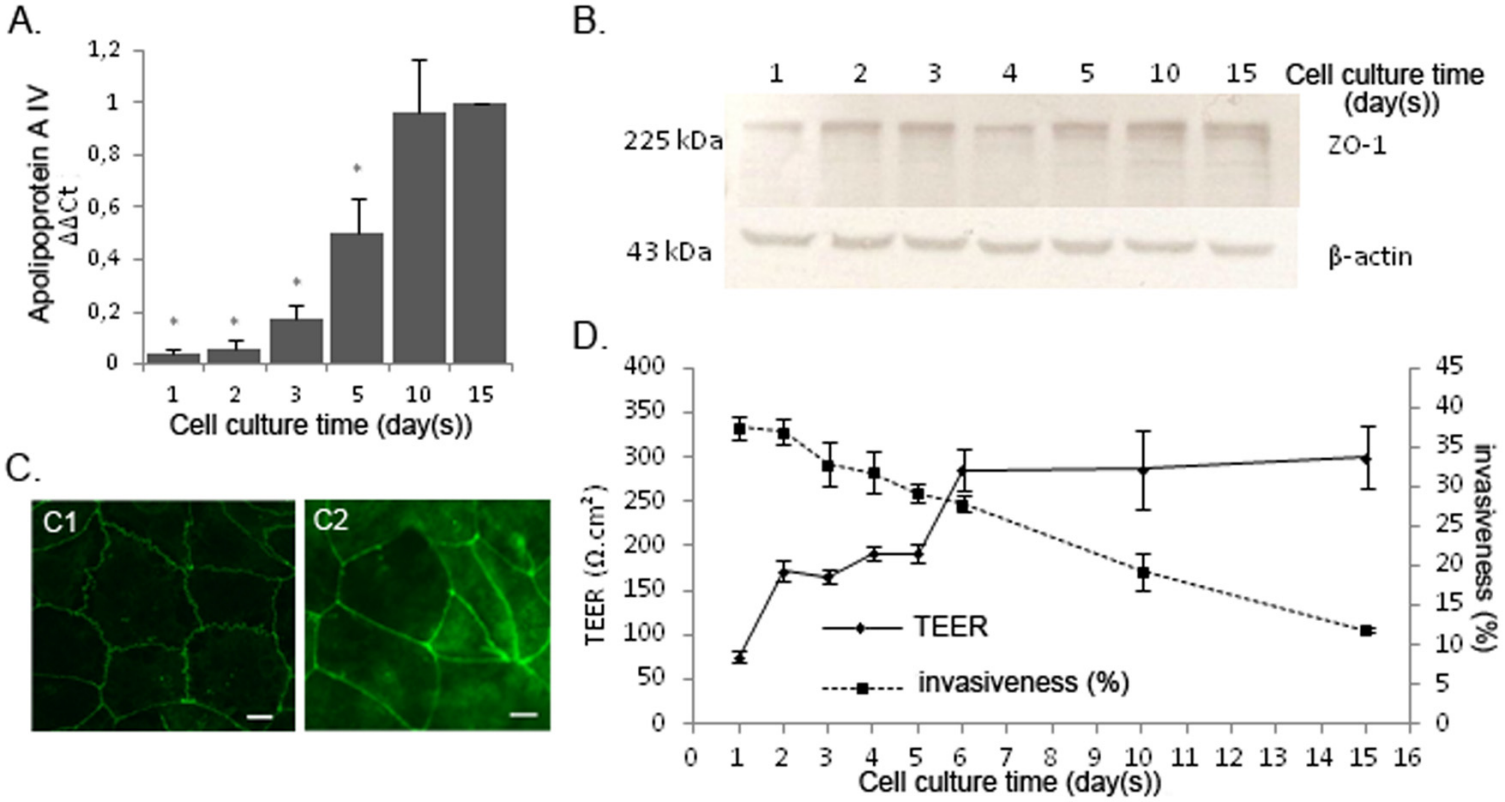
A decrease in the invasiveness of *Candida albicans* into Caco-2 cells parallels the differentiation of intestinal epithelial cells. A. The differentiation level of Caco-2 cells was evaluated by quantifying the GAPDH- normalized expression of the Apolipoprotein A-IV gene at different days post-seeding. The expression level of the Apolipoprotein A-IV gene at day 15 post-seeding was used as the reference expression level of fully differentiated Caco-2 cells. The gene expression levels of Apolipoprotein A-IV (ΔΔCt) were statistically lower after 1, 2, 3 and 5 days post-seeding than at day 15 as a result of incomplete differentiation (*p < 0.05, Mann–Whitney test). For each time point, data are presented as the mean of four independent biological replicates. B. The expression level of the TJ constitutive protein ZO-1 increases throughout the differentiation of Caco-2 cells. ZO-1 protein expression was quantified by SDS page and blotting compared to β-actin as a housekeeping protein. C. Immuno-staining of the ZO-1 protein at day 2 shows a heterogeneous and rough, zigzag distribution, indicating immature TJ (C1) whereas ZO-1 staining 15 days post-seeding shows a thin and homogeneous distribution of ZO-1 at the inter-cell membrane of adjacent enterocytes, reflecting the presence of mature TJ (C2) (Scale bar: 10μm). D. Caco-2 cell differentiation increased throughout the culture as highlighted by the increase in TEER measurements (Ω.cm^2^) (full line), which correlated with a decrease in *C*. *albicans* invasion (dotted line). For each time point, Caco-2 cells were incubated with *C*. *albicans* SC5314 for 2 h, after which the number of *Candida* cells partially internalized into enterocytes was determined using a differential fluorescence assay, as described in the experimental procedures section. Results show the mean ± standard deviation of at least three independent experiments for each of which, 300 *C*. *albicans*’ interacting cells were checked for adherence to and/or invasion into Caco-2 cells (*p < 0.05, Kruskal and Wallis’ test).

To allow the access of *C*. *albicans* to the basolateral side of enterocytes, a first series of experiments was conducted using Caco-2 cells at different stages of differentiation and consequently presenting Tight Junctions (TJ) at different stages of maturation. The kinetics of differentiation of Caco-2 cells was first monitored by measuring gene expression of the Apolipoprotein A-IV (apoA-IV), a marker of IEC differentiation, by RT-qPCR at days 1, 2, 3, 5, 10 and 15 post-seeding (Fig 1A) [26, 27]. Whereas apoA-IV gene expression was significantly detectable at day 5 post-seeding, complete differentiation of Caco-2 cells was obtained after day 10 post-seeding, when gene expression of the apoA-IV marker reached a plateau.

Zonula Occludens-1 (ZO-1) protein is a major constitutive protein of the TJ, and is frequently used to characterize the structural function of the TJ. Detection of ZO-1 protein by western blot showed the presence of ZO-1 protein in Caco-2 cells as early as day 1 post-seeding but in smaller amounts than at day 15 post-seeding. From day-1 onwards, the amount of total cellular ZO-1 protein increased throughout the differentiation process (Fig 1B). Similarly, immunofluorescence microscopy of ZO-1 protein (Fig 1C) at day 15 post-seeding revealed a thin and homogeneous line of staining at the interface of adjacent enterocytes, as a result of the complete maturity of TJ (Fig 1C-C2) [28]. In contrast at day-2 post-seeding, microscopy showed heterogeneous, rough, zigzag staining reflecting the immaturity of the TJ (Fig 1C-C1) [29].

In parallel, the Trans-Epithelial Electrical Resistance (TEER) of Caco-2 cells cultured on polycarbonate inserts was measured at days 1, 2, 3, 4, 5, 6, 10 and 15 post-seeding (Fig 1D) as a marker of the impermeability of the IEC monolayer, which specifically correlates with the development of efficient TJ [24]. TEER increased progressively throughout the differentiation process until it reached a plateau at day-6 post-seeding. These data suggested that TJ ensured the impermeability of the Caco-2 cell monolayer from day 6 post-seeding onwards. Simultaneously to the kinetics of TJ maturation, the invasion of Caco-2 cells by *C*. *albicans* SC5314 was assayed at days 1, 2, 3, 4, 5, 6, 10 and 15 post-seeding (Fig 1D). A progressive decrease in the percentage of fungal invasiveness into enterocytes was observed throughout the course of Caco-2 cell differentiation, ranging from 37.5% at day 1 post-seeding to 11.8% at day 15 post-seeding. Altogether, these data support the view that the efficiency of Caco-2 cell invasion by *C*. *albicans* decreases in parallel with epithelial cell differentiation, suggesting that TJ play a protective role in limiting the invasiveness of the fungus.

### Mature TJ play a protective role in intestinal cells by limiting the invasiveness of *C*. *albicans*

**Fig 2:**
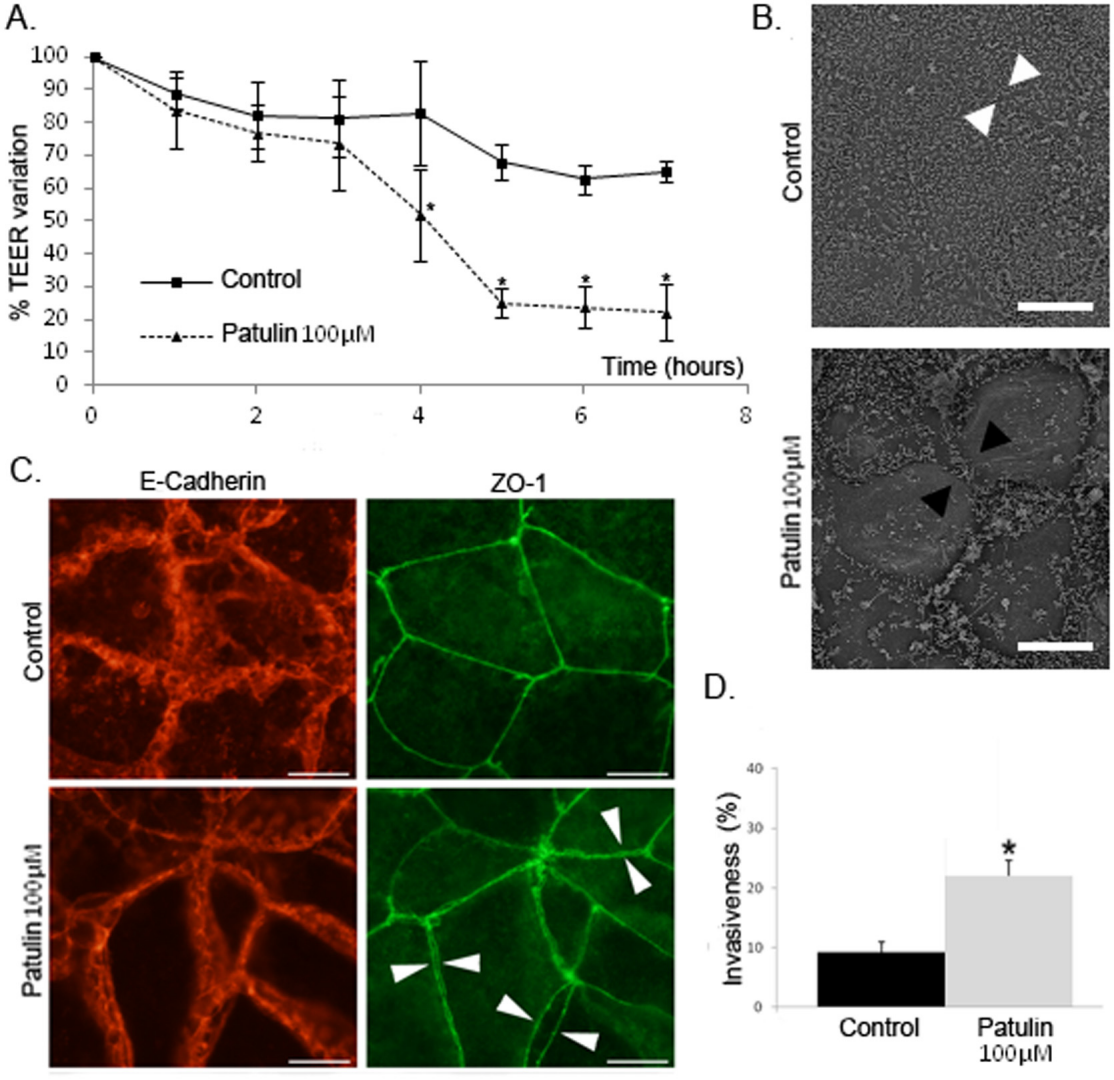
TJ alteration by patulin treatment induces an increase in *C*. *albicans* invasion into differentiated Caco-2 cells. A. Modulation of TJ was induced in Caco-2 cells by treatment with patulin 100μM for 4 hours. TEER measurements showed a significant increase in tissue permeability of Caco-2 cells incubated with DMEM with patulin 100μM (dotted line), as compared with cells incubated with DMEM alone (full line). TEER remained stable when patulin was removed. (*p<0.05, Mann-Whitney’s test). B. Released cellular junctions (white arrow heads) were observed in Caco-2 cells treated with patulin 100μM as compared to control cells (black arrow heads) (Scale bar: 10 μm). C. Treatment with patulin specifically alters TJ integrity. After treatment with patulin 100μM, co-immunostaining of both E-Cadherin (left column) and ZO-1 (right column) showed a steady state membrane localization of E-Cadherin whereas the distribution of ZO-1 highlighted “double membrane” staining of adjacent enterocytes (arrow heads), illustrating the disruption of TJ integrity, but not the integrity of the E-cadherin containing junctions (*i*.*e*. Adherens junctions) (Scale bar: 10 μm). D. The invasiveness of *C*. *albicans* was statistically greater in patulin pre-treated Caco-2 cells than in untreated Caco-2 cells. After 4h incubation with patulin 100μM or with culture medium alone (Control), Caco-2 cells were infected with *C*. *albicans* SC5314 for 2 hours after which the number of *Candida* cells partially internalized into enterocytes was determined using a differential fluorescence assay as described in the experimental procedures section. Results show the mean ± standard deviation of at least three independent experiments for each of which, 300 *C*. *albicans* interacting cells were checked for adherence to and/or invasion into Caco-2 cells (*p < 0.05, Kruskal and Wallis’ test).

A second series of experiments was conducted to determine the impact of altering TJ integrity at mature stages of enterocyte differentiation using patulin, a mycotoxin that has been shown to specifically alter the integrity of TJ by inducing the phosphorylation of ZO-1 and consequently its degradation [23]. TEER values in a monolayer of differentiated Caco-2 cells treated with patulin 100 μM for 4 hours were significantly lower (57.6%) than those in control cells (*i*.*e*. differentiated Caco-2 cells without pre-treatment) (Fig 2A). This phenomenon was not reversible since replacement of the medium after the pre-treatment period with fresh medium without patulin 100 μM did not lead to an increase in the TEER to values comparable to the control (Fig 2A). To exclude cellular toxicity of the patulin 100 μM pre-treatment, cytotoxicity assays were performed using differentiated Caco-2 cells incubated with DMEM containing patulin 100 μM for 8 hours before staining the cells with the dead-cell marker Sytox Orange Nucleic Acid Stain. No significant cytotoxicity of patulin 100 μM was observed in Caco-2 cells as well as in *Candida* cells since hyphae formation in pre-treated Caco-2 cells was similar to that in Caco-2 control cells. Consequently, we concluded that the stable decrease in TEER values was a consequence of the irreversible disorganization of TJ integrity by patulin. To determine the precise effect of patulin on cell junction integrity, patulin 100 μM pre-treated Caco-2 cells and control cells were examined by microscopy. First, scanning electron microscopy (SEM) images highlighted darker lines between adjacent enterocytes in pre-treated IEC than in control IEC, suggesting the presence of wider inter-cellular spaces in the former as a consequence of the release of cellular junctions (Fig 2B). To determine which junctions were affected, co-immunostaining of both Adherens junctions (AJ) and TJ was performed by staining E-cadherin and Z0-1, respectively (Fig 2C). In patulin 100 μM pre-treated Caco-2 cells, ZO-1 staining revealed the presence of double labelling between adjacent enterocytes, suggesting the opening of inter-cellular TJ. In contrast, staining of the E-cadherin protein in patulin 100 μM pre-treated IEC was comparable to that of control IEC, thus confirming the stability of AJ under these conditions and the specific effect of patulin on TJ without altering AJ. The relevance of these observations was established by impairing cellular junctions with EGTA pre-treatment, which resulted in disorganization of the intracellular space at both the TJ and AJ level (S2 Fig). Invasion assays were then performed using differentiated Caco-2 cells pre-treated for 4 hours with patulin 100 μM before infection with *C*. *albicans*. A significant increase in the invasiveness of *C*. *albicans* into patulin 100 μM pre-treated cells was observed after two hours of contact (22.0%) compared with control cells (9.3%) (Fig 2D). Taken together, these data confirmed the importance of TJ integrity in limiting the invasion of *C*. *albicans* into enterocytes.

### Endocytosis contributes to the invasion of *C*. *albicans* into intestinal cells displaying altered TJ

**Fig 3:**
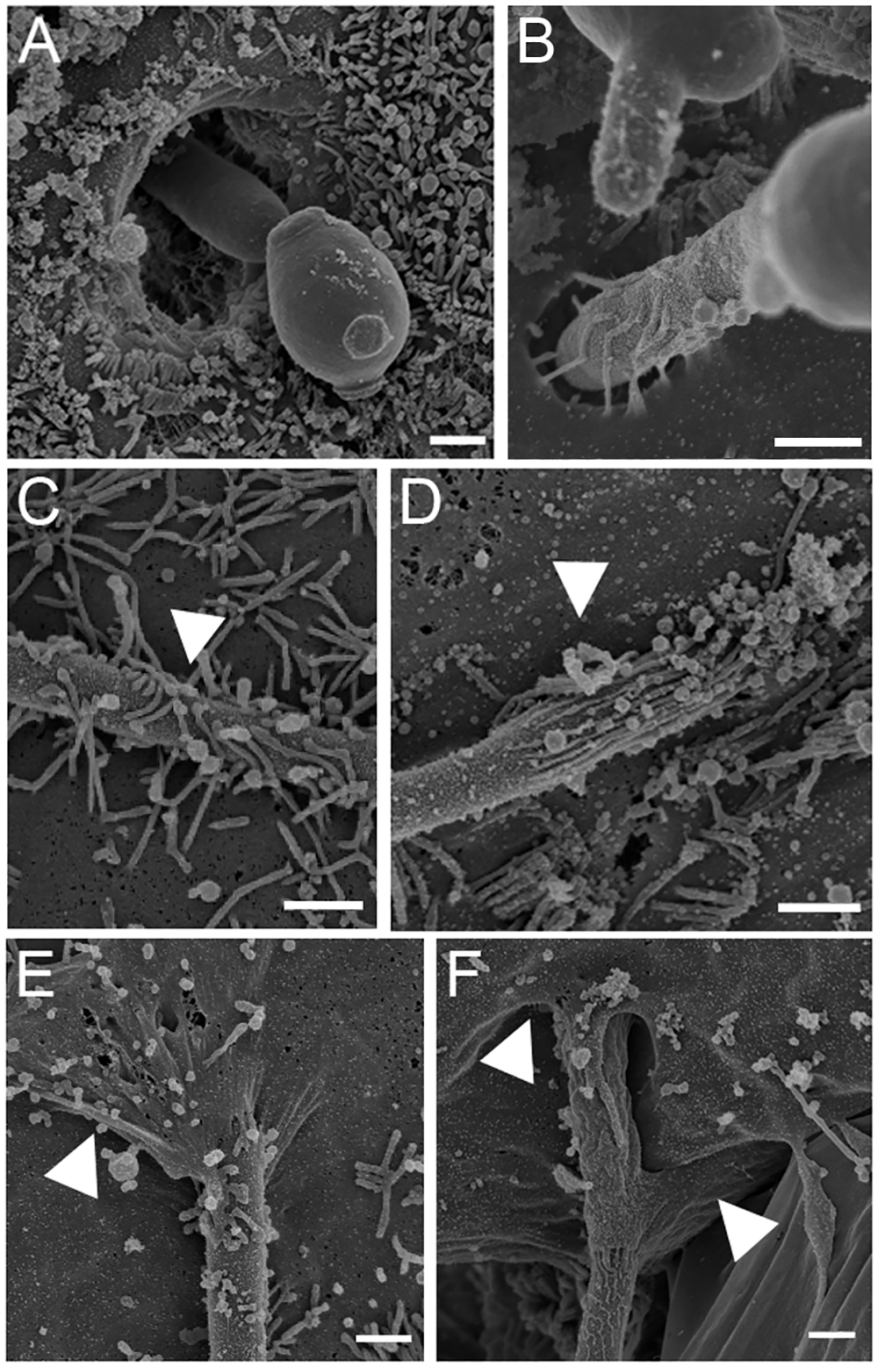
Interactions of *C*. *albicans* with enterocytes after TJ alteration with patulin. SEM micrographs of Caco-2 cells pre-treated with patulin 100μM for 4 h and then incubated with *C*. *albicans* SC5314 for 2 h. A and B. SEM micrograph showing hyphal *Candida* cells actively penetrating enterocytes. This was observed in both Control (A) and patulin 100μM pre-treated cells (B). C and D. SEM micrograph showing microvilli-like structures intimately attached to a germinated *C*. *albicans* cell. Arrowhead indicates the membrane ruffling closely localized to the hypha, which was observed in both Control (C) and patulin 100μM pre-treated cells (D). E and F. *C*. *albicans* hyphal cells locally engulfed by enterocyte cell membranes (arrow heads). Such interactions were observed only in Caco-2 cells pre-treated with patulin 100μM (Scale bar: 1μm).

To determine which cellular mechanisms facilitate the invasion of *C*. *albicans* into enterocytes in the absence of TJ, SEM was used to observe interactions between *C*. *albicans* and differentiated Caco-2 cells pre-treated with patulin 100μM or not (*i*.*e*. control IEC). These observations showed microvilli-structures intimately attached to the hyphal *Candida* cells (Fifs 3C and 3D) as well as depressions on the epithelial surface at the site of penetration of *C*. *albicans* hyphae, suggesting an active penetration process in both patulin 100μM pre-treated cells (Fig 3A) and control IEC (Fig 3B), as previously described [8]. However, membrane protrusions surrounding *C*. *albicans* hyphae were observed in patulin 100μM pre-treated cells only, strongly suggesting that endocytosis is a possible mechanism of internalization of *C*. *albicans* in these conditions (Heads of arrows, Fig 3E and 3F).

**Fig 4:**
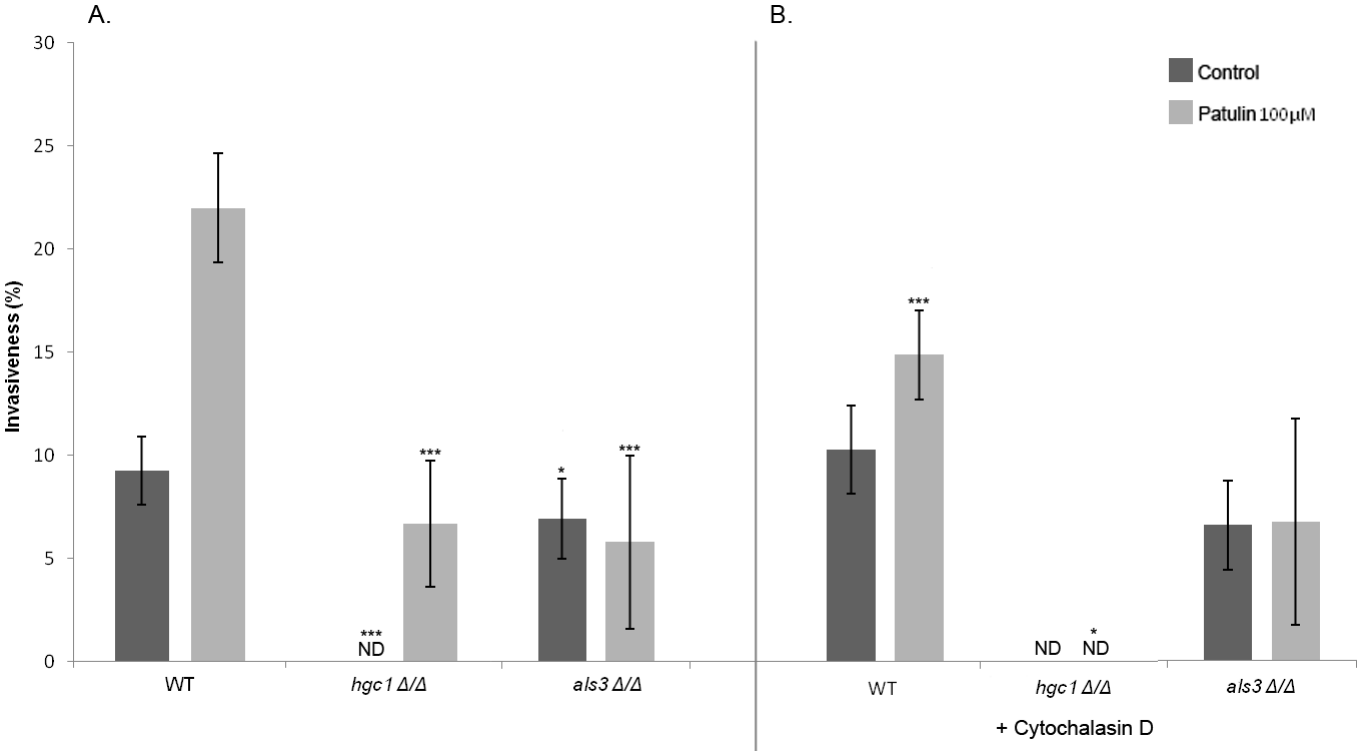
Contribution of *C*. *albicans* invasins to internalization of the fungus by Caco-2 cells pre-treated or not with patulin 100μM. Caco-2 cells pre-treated or not with patulin 100μM were infected with *C*. *albicans* for 2 h after which the number of *Candida* cells partially internalized in enterocytes was determined. The relative contribution of active penetration and endocytosis in the internalization of the fungus was evaluated using Cytochalasin D treatment and *C*. *albicans* mutants (*hgc1Δ/Δ* and *als3Δ/Δ*). Invasiveness corresponded to the percentage of hyphae partially internalized amongst adherent hyphae. Results show mean ± SD of at least three independent experiments counting 300 yeasts for each (*p < 0.05, Kruskal and Wallis’ test) (*** p < 0.001, Anova test). ND: not detected. A. (no CytD treatment) The invasiveness of *C*. *albicans* invasins mutants (*hgc1Δ/Δ*, *als3Δ/Δ)* by enterocytes pre-treated or not with patulin 100μM was compared to that measured in the wild-type strain SC-5314 (WT). B. (CytD treatment) The invasiveness was compared with that measured in the same conditions but in the absence of treatment with cytochalasin D.

To confirm these observations, we therefore investigated the effect of cytochalasin D (CytD) and amiloride, *i*.*e*. inhibitors of endocytosis, on the internalization of *C*. *albicans* by differentiated Caco-2 cells pre-treated with patulin 100μM or not (control IEC). After 2 hours of contact, CytD 0.5 μM caused a 34.5% (p<0.0001) reduction in the internalization of *C*. *albicans* by differentiated patulin 100μM pre-treated Caco-2 cells (Fig 4, see WT: 22.00% w/o CytD 0.5 μM *versus* 14.33% with CytD 0.5 μM) but not by control IEC. In the same conditions, amiloride 0.5 mM significantly reduced internalization of *C*. *albicans* into differentiated Caco-2 cells pre-treated with patulin 100μM (22.00% w/o amiloride *versus* 15.17% with amiloride 0.5 mM) but not into control IEC. Altogether, these data suggest that endocytosis is a possible mechanism of internalization of *C*. *albicans* by enterocytes when TJ are altered (*i*.*e*. in differentiated Caco-2 cells pre-treated with patulin 100μM), occurring through zipper-like and macropinocytosis-like mechanisms.

### Only hyphal *C*. *albicans* cells can be endocytosed by enterocytes displaying altered TJ

The internalization of different *C*. *albicans* mutants defective for genes known to be involved in hyphae formation and invasion into epithelial cells was studied (Fig 4). Since for each of the mutants tested, the invasiveness of the parental strain and the revertant were shown to be not different from those of the WT strain *C*. *albicans* SC5314 (S3 Fig), the WT strain was presented as the control in all the experiments below (Fig 4).

Internalization of the *C*. *albicans hgc1Δ/Δ* mutant was further investigated. Indeed, *HGC1* is a hypha-specific gene which is essential for morphogenesis. Deletion of *HGC1* abolishes hyphal growth but preserves expression of different epitopes specific to the surface of the hyphae. Since our previous observations suggest the importance of *C*. *albicans* hyphae in both endocytosis and active penetration processes, this mutant provided the opportunity to test the ability of enterocytes to internalize *C*. *albicans* in a morphological form not able to produce hyphae but expressing epitopes of the filamentous form. Invasion of the *hgc1Δ/Δ* mutant into non-treated cells was not detected, whereas it reached 6.7% when interacting with differentiated Caco-2 cells pre-treated with patulin 100μM (Fig 4A). These observations confirmed the importance of active penetration in the invasion of *C*. *albicans* into non pre-treated enterocytes since the *hgc1Δ/Δ* mutant was not able to produce hyphae. However, the residual invasion of *C*. *albicans* into patulin 100μM pre-treated Caco-2 cells suggests that *C*. *albicans hgc1Δ/Δ* expresses at its surface various hypha-specific ligands putatively involved in invasiveness. As *C*. *albicans hgc1Δ/Δ* is not able to produce hyphae (*i*.*e*. not able to invade epithelial cells *via* active penetration), these data suggest that *C*. *albicans hgc1Δ/Δ* is able to invade enterocytes through an endocytosis mechanism, when TJ are altered. In addition, no invasion of killed yeast forms of *C*. *albicans* SC5314 into Caco-2 cells pre-treated or not with patulin 100μM was detectable, corroborating the importance of hypha-specific epitopes in both active penetration and endocytosis mechanisms, as described elsewhere [8].

Phan et *al*. reported that the binding of *C*. *albicans* adhesin Als3 to E-cadherin could trigger endocytosis of the fungus by oral epithelial cells [11]. Als3 is a fungal surface protein, preferentially localized at the surface of hyphal forms. It acts as an adhesin and facilitates invasion [11]. In order to determine the role of Als3 in the internalization of *C*. *albicans* by enterocytes, we tested the invasiveness of *C*. *albicans* a*ls3Δ/Δ* mutant into Caco-2 cells displaying or not pharmacologically altered TJ. Even though this mutant does not show any defect in hyphal formation, decreased internalization of *C*. *albicans als3Δ/Δ* compared to the WT strain was observed in both control IEC (WT: 9.27% vs *als3Δ/Δ*: 6.67%; p<0.05) and differentiated Caco-2 cells pre-treated with patulin 100μM (WT: 22.00% vs *als3Δ/Δ*: 5.60%; p<0.001; Fig 4A). These observations confirmed previous observations reporting the importance of Als3 in active penetration of the fungus into epithelial cells [11]. In addition, the internalization of *C*. *albicans als3Δ/Δ* into Caco-2 cells pre-treated with patulin 100μM in addition to CytD 0.5 M was not statistically different from internalization of the same mutant into Caco-2 cells pre-treated with patulin 100μM only (Fig 4B). The observation that blocking induced endocytosis in the absence of Als3 had no significant effect on fungal invasion confirms previous studies reporting that Als3 is crucial in mediating induced endocytosis into epithelial cells [9, 11].

### Interaction with E-cadherin is not necessary for endocytosis of *C*. *albicans* into enterocytes

**Fig 5:**
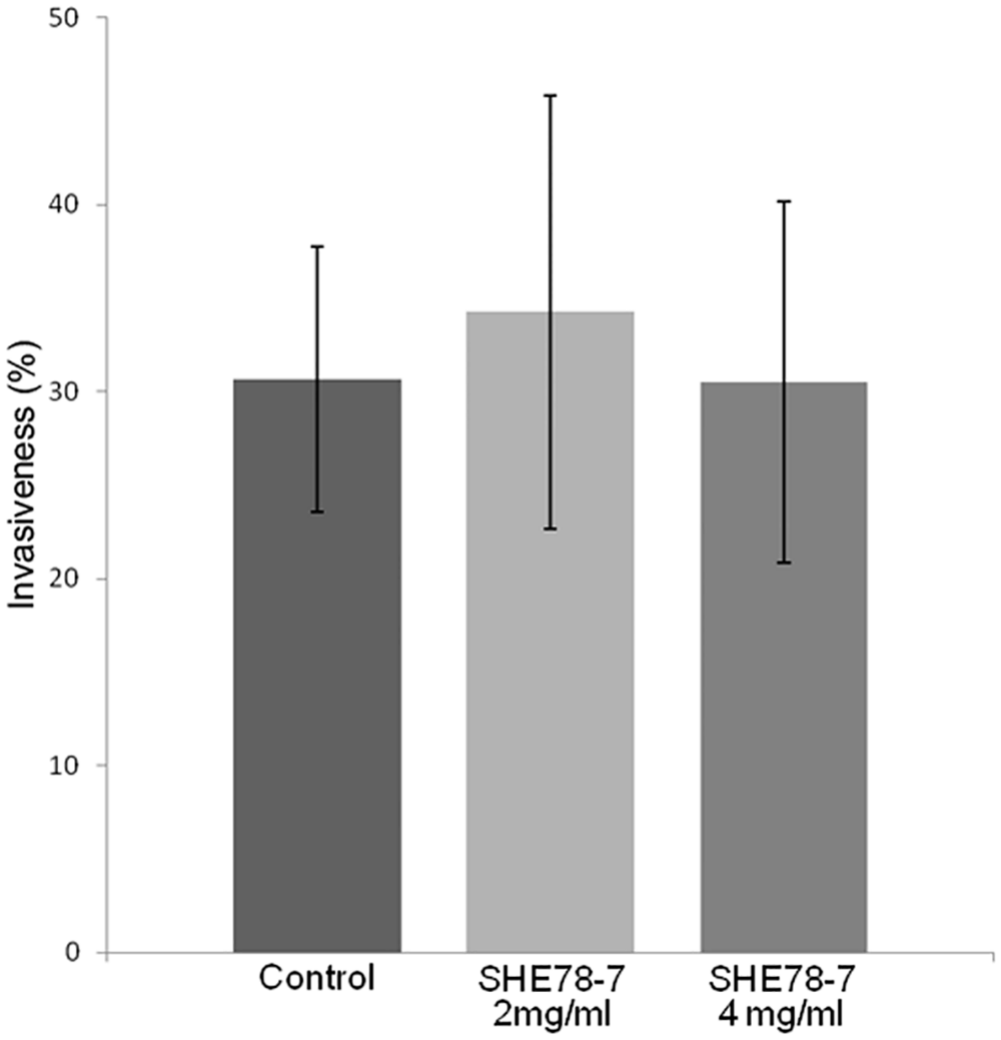
E-cadherin immunoblocking did not modify the invasiveness of *C*. *albicans* into Caco-2 cells lacking TJ. Differentiated Caco-2 enterocytes pre-treated with 100μM patulin (patulin) were incubated with *C*. *albicans* in the presence of 2 mg/ml and 4 mg/ml of the blocking antibody anti-E-cadherin SHE78-7. No significant differences were observed between the conditions tested.

It has been demonstrated that *C*.*albicans* hyphae bind to N-cadherin of endothelial cells and to E-cadherin of oral epithelial cells, thus triggering endocytosis of the fungus [11, 30]. Concerning enterocytes, E-cadherin is localized at the Adherens junctions, immediately under TJ. We hypothesized that endocytosis of *C*. *albicans* by enterocytes could be the consequence of the access of *C*. *albicans* hyphae to E-cadherin when TJ are altered. To assess this question, we used the mouse monoclonal anti-E cadherin antibody SHE78-7 to block the interaction of *C*. *albicans* hyphae to E-cadherin in enterocytes with altered TJ. The blocking effect of the monoclonal antibody was first validated using the *L*. *monocytogenes* model of invasion into Caco-2 cells. Indeed, it has already been shown that Caco-2 cells could internalize these pathogenic bacteria *via* an endocytosis mechanism involving the interaction of Internalin A, a surface molecule of the bacteria, with E-cadherin [31]. Invasion assays were further performed by incubating *L*. *monocytogenes* with Caco-2 cells pre-treated with 2 or 4mg/mL of the mouse monoclonal anti-E cadherin antibody SHE78-7. A strong decrease in the invasion of the bacteria was observed at both concentrations, confirming the blocking effect of the SHE78-7 antibody (S4 Fig). We then tested the effect of the blocking monoclonal antibody on the invasion of *C*. *albicans* into Caco-2 cells with altered TJ (*i*.*e*. with accessible E-cadherin). The mouse monoclonal anti-E cadherin antibody at a concentration of 2 and 4 mg/mL was added to differentiated Caco-2 cells pre-treated with patulin 100 μM for 2 hours (Fig 5). No significant difference was observed for invasion of the fungus, suggesting that E-cadherin was not involved in endocytosis of *C*. *albicans* hyphae into Caco-2 cells with altered TJ.

## Discussion

In this study, we demonstrated that endocytosis of *C*. *albicans* was a possible mechanism for the internalization of the yeast into intestinal epithelial cells (IEC) displaying altered TJ.

Dalle et *al* previously reported that epithelial oral cells of the TR146 cell line were able to internalize *C*. *albicans* through an endocytic processes involving a zipper-like mechanism and a macropinocytosis-like process since endocytosis of *C*. *albicans* was drastically inhibited by cytochalasin D 0.5 M (a specific inhibitor of actin polymerisation) and amiloride 0.5 mM (a specific inhibitor of macropinocytosis) [8]. During the interaction of *C*. *albicans* with oral epithelial cells, endocytosis of the yeast is triggered by the binding of Als3, an adhesin produced at the surface of *C*. *albicans* hyphae, to E-cadherin, which is expressed at the surface of oral epithelial cells. This recognition is possible since the molecular structure of Als3 mimics the host-cell receptor of cadherins [11]. Enterocytes also express E-cadherin, the main protein of Adherens Junctions (AJ). Franck et *al* reported that *C*. *albicans* was able to cleave E-cadherin, leading to disruption of the interactions between adjacent enterocytes and a loss of cellular integrity of the enterocyte layer [32]. This suggests that *C*. *albicans* was able to interact with E-cadherin in enterocytes. However, this phenomenon was described at the late stages of the infection process (12-24h post-infection) and has not yet been associated with internalization of the fungus through endocytosis. Finally, though Weide et *al*. suggested that endocytosis was a possible route of internalization of *C*. *albicans* by intestinal cells [33], this mechanism has not yet been demonstrated.

The gut mucosa is composed of differentiated epithelial cells arranged in a monolayer of enterocytes. These are highly polarized cells thanks to their contact with the basement membrane and with adjacent enterocytes [34]. When the contact of enterocytes with components of the extra-cellular matrix and the basement membrane initiates the differentiation process of epithelial cells, TJ connect adjacent enterocytes in the late steps of differentiation, and thus contribute to the impermeability and robustness of the intestinal barrier. Consequently, TJ are the most apical junctions, and limit the passage of molecules, nutrients and micro-organisms from the apical to the basolateral side, thus ensuring perfect impermeability of the intestinal epithelial layer. AJ, which are located immediately below the TJ, form an adhesive belt and ensure the architectural cohesion of the epithelium.

In this study, we hypothesized that endocytosis of *C*. *albicans* was not observed during the early steps of its interaction with enterocytes because of the apical localization of TJ, which limits access of the fungus to E-cadherin at the AJ level.

To assess the role of TJ during interactions with intestinal epithelial cells, we first evaluated the critical timing of functional TJ formation by studying the kinetics of Caco-2 cell differentiation using different markers. The data we obtained demonstrated that structural and functional maturity of TJ paralleled the differentiation kinetics of Caco-2 cells.

The invasion of *C*. *albicans* into Caco-2 cells at different stages of differentiation was then evaluated. Interestingly, we noted that invasion into Caco-2 cells decreased throughout the course of Caco-2 cell differentiation, suggesting that TJ may contribute to limiting the invasion of enterocytes by the fungus.

Many compounds are able to disorganize the structure and function of TJ by providing either a simple opening of the TJ or by complete destruction of all of the constitutive proteins of intercellular junctions [35]. Among these compounds, the calcium chelator Ethylene Glycol Tetra-acetic Acid (EGTA) and patulin were tested because they have no inhibitory effect on yeast growth. However, because inter-cellular junctions mainly involve Ca^++^-dependent binding proteins, extracellular depletion of calcium using EGTA alters the structure and function of both TJ and other inter-cellular junctions, including AJ [36, 37] (S2 Fig). In contrast, patulin, a mycotoxin that is a secondary metabolite of fungi belonging to the genera *Aspergillus*, *Penicillium* or *Byssochlamys*, has been reported to specifically alter the TJ. This was confirmed in our experiments (Fig 2 and S2 Fig) [23]. Indeed, patulin is mainly found in fruits, especially apples, and was reported to increase epithelial permeability of the intestinal barrier by modulating the TJ through occludin proteolysis and reduction of ZO-1 protein levels [23, 38, 39]. The specific effect of patulin on TJ integrity was confirmed in our experiments (Fig 2 and S2 Fig) as reported elsewhere [38, 39]. Finally, further experiments were conducted using patulin as the pharmacological agent to specifically alter TJ integrity.

A drastic and significant increase in the invasion of *C*. *albicans* into Caco-2 cells pre-treated with patulin 100μM was observed, thus providing evidence that TJ play a crucial role in limiting invasion of *C*. *albicans* into intestinal cells. These observations corroborate other studies that have reported abnormal bacterial translocation across the epithelial barrier via the paracellular route *in vitro* [40] and *in vivo* [41–43] as a consequence of an increase in the paracellular permeability related to TJ disruption.

Interestingly, the fact that oral epithelial cells do not have TJ may explain the difference in *C*. *albicans* invasiveness depending on the epithelial cell type as previously reported [8]. Indeed, we showed that adherence, invasion and cellular damage were higher in oral epithelial cells than in differentiated intestinal cells. In the present study, pharmacologically altering the TJ drastically increased the invasion of *C*. *albicans* into differentiated Caco-2 cells, suggesting that the absence of TJ facilitates invasion of the fungus into oral epithelial cells. Finally, the increase in the invasiveness of *C*. *albicans* into intestinal cells displaying altered TJ raises the question of whether active penetration and/or endocytosis could contribute to internalization of the fungus in these conditions.

Invasion assays were repeated in the presence of inhibitors of endocytosis. Cytochalasin D, an inhibitor of F-actin polymerization [44] and amiloride, a specific inhibitor of macropinocytosis [20], were used because they inhibit endocytosis of *C*. *albicans* by oral TR146 cells [8]. A decrease in the internalization of *C*. *albicans* was observed when differentiated Caco-2 cells displaying altered TJ were pre-treated with both cytochalasin D and amiloride. These results suggest that differentiated Caco-2 cells are able to internalize *C*. *albicans* through endocytosis when TJ are pharmacologically altered. This was supported by SEM observations of *C*. *albicans* interacting with differentiated Caco-2 cells pre-treated with patulin 100μM (Fig 3C and 3D). These observations highlighted membrane protrusions forming a sleeve around *C*. *albicans* hyphae. In addition, the inhibitory effect obtained with amiloride suggests that macropinocytosis is an endocytosis-like process that contributes to internalization of the fungus into Caco-2 cells with pharmacologically altered TJ. These data corroborate other findings reporting macropinocytosis as a crucial mechanism for endocytosis of microorganisms such as *Escherichia coli* [45, 46] or viruses [47].

However, a small percentage of *C*. *albicans* were still able to invade CytD 0.5 M pre-treated Caco-2 cells displaying altered TJ, suggesting that, in addition to actin-mediated endocytosis, *C*. *albicans* is able to invade Caco-2 cells displaying altered TJ through active penetration.

To rule out the active penetration of the fungus in the invasion process, endocytosis inhibition assays were further conducted using *C*. *albicans hgc1Δ/Δ*, a mutant not able to produce hyphae but able to express different proteins normally associated with *C*. *albicans* hyphae. As a result of the lack of hyphae formation by this mutant, a crucial feature for active penetration of *C*. *albicans*, we were able to show that hypha-specific proteins were necessary for endocytosis of *C*. *albicans* into differentiated Caco-2 cells displaying altered TJ. These data confirmed that endocytosis of *C*. *albicans* by differentiated Caco-2 cells is an active process from the host cells and is associated with the presence of hypha-specific proteins of *C*. *albicans*. Finally, our findings corroborate the view that the filamentous form is a crucial attribute for *C*. *albicans* invasion into epithelial cells *via* both routes (*i*.*e*. active penetration and endocytosis), thus confirming previous observations [8].

Among these hypha-specific proteins, Als3 has previously been reported to play a key role in multiple processes including adherence to host cells and host-cell invasion [48]. It has been shown that Als3 is a key invasin necessary for the internalization of *C*. *albicans* into epithelial cells via both routes (*i*.*e*. active penetration and endocytosis [9]). In addition, the binding of Als3 to E-cadherin has been reported to trigger endocytosis of the fungus by oral epithelial cells [11]. In this work, we were able to provide evidence that Als3 contributes to endocytosis of the fungus into enterocytes displaying altered TJ. However, neutralizing the interaction of Als3 with E-cadherin by using an anti-E-Cadherin antibody did not modify internalization of *C*. *albicans als3Δ/Δ* into differentiated Caco-2 cells displaying altered TJ. This blocking antibody approach has limitations (lack of sensitivity, zone phenomena, wrong epitope targeting…) and other experiments using an siRNA approach or KO Caco-2 cells for the constitutive expression of E-cadherin would be necessary to confirm these observations. Nevertheless, the efficacy of the SHE78-7 antibody in blocking E-cadherin was confirmed in our experiments using the *Listeria monocytogenes* model, another micro-organism model, in which endocytosis into enterocytes was reported to involve interactions between Internalin A, a surface molecule of the bacteria, and E-cadherin [31]. Finally, our data support the view that Als3 contributes to the internalization of *C*. *albicans* into differentiated Caco-2 cells displaying altered TJ by an endocytic process that does not involve the interaction of Als3 with E-cadherin.

Interestingly, Zhu et *al*. recently reported that inhibition of E-cadherin function only partially reduced endocytosis of *C*. *albicans* by epithelial oral cells, suggesting that other host cell receptors may be involved in triggering endocytosis by Als3 [49]. They then showed that EGFR and HER2 play a major role in endocytosis of the fungus by oral epithelial cells. Whether these receptors participate in endocytosis of the fungus into differentiated Caco-2 cells displaying altered TJ remains to be determined.

In addition, according to Zhu et *al*. [49], other invasins could be involved in the internalization of *C*. *albicans* by oral cells. Indeed, the hypha-specific invasin Ssa1 has been shown to contribute to endocytosis of *C*. *albicans* by oral epithelial cells. Consequently, investigating the role of Ssa1 in the endocytosis of *C*. *albicans* by intestinal cells displaying altered TJ would be of interest to better understand the mechanisms by which *C*. *albicans* triggers its own endocytosis in these host cell conditions.

In conclusion, we report here for the first time that tight junctions play a crucial role in limiting the invasion of the human pathogen *C*. *albicans* at the early stages of its interaction with the intestinal barrier. Moreover, we provide evidence that, as well as active penetration of the fungus into intestinal cells, altering the TJ enables internalization of *C*. *albicans* by enterocytes. Finally, these observations support the view that exogenous factors or pathological conditions that disturb the integrity of tight junctions could promote invasion of *C*. *albicans* through the intestinal barrier.

## Supporting Information

S1 Fig Effect of the various treatments used in this study upon hyphal development. Filamentation assay were conducted with *C*. *albicans* SC5314 incubated in serum for 4 hours at 37°C in the presence of 100 μM patulin, 0.5μM cytochalasin or both and compared with yeasts incubated in the same conditions without any drugs. No effect of the drugs was observed on the number and the length of the hyphae formed (Statistics: Anova test).
(TIF)

S2 Fig Characterization of the effect of EGTA treatment on differentiated Caco-2 cells. Differentiated Caco-2 cells were pre-treated with 2.5mM EGTA (Sigma-Aldrich^®^, Saint-Quentin Fallavier, France), a calcium chelator, in DMEM without calcium for 30 min at 37°C, 5% CO_2_. The medium was then replaced with fresh DMEM without calcium in order to maintain TJ in an open conformation. A. Fully released cellular junctions were observed in Caco-2 cells treated with 2.5 mM EGTA (Scale bar: 10 μm). B. Treatment with patulin specifically altered TJ integrity, whereas EGTA induced complete disruption of the cellular junctions (*i*.*e*. Adherens junctions) illustrated by the relocation of staining of both E-cadherin and ZO-1 (Scale bar: 10 μm).
(TIF)

S3 Fig Invasiveness of parental and revertant *C*. *albicans* invasin mutants compared with the reference SC5314 strains (WT). Caco-2 cells were infected with *C*. *albicans* for 2 hours after which the number of *Candida* cells partially internalized into enterocytes was determined using a differential fluorescence assay as described in the experimental procedures section. Results show the mean ± standard deviation of at least three independent experiments for each of which, 300 *C*. *albicans*’ interacting cells were checked for adherence to and/or invasion into Caco-2 cells (Statistics: Kruskal and Wallis’ test).
(TIF)

S4 Fig E-cadherin immunoblocking modified the invasiveness of *L*. *monocytogenes* strain ScottA into Caco-2 cells. Differentiated Caco-2 enterocytes were inoculated with *Listeria monocytogenes* strain ScottA at a MOI of 100 in the presence of 2 and 4 mg/ml of the blocking antibody anti-E-cadherin SHE78-7. Significant differences were observed between the various conditions (*p<0.05, Anova test). **Bacterial growth conditions and bacterial invasion assays:** All experiments performed with *Listeria monocytogenes* (*L*. *monocytogenes)* were carried out with *L*. *monocytogenes* strain ScottA (Institut Pasteur Collection, Paris, France) [1]. Bacteria were grown routinely on blood agar plates. For infection experiments, bacteria were grown overnight in Brain Heart Infusion (BHI) at 37°C. *L*. *monocytogenes* cells were then diluted in fresh BHI and grown for 2 to 3 hours at 37°C to obtain an optical density between 0.20 and 0.30 at 600 nm. Bacterial suspensions were then adjusted to the desired concentration in DMEM. Adhesion and invasion assays were performed using a multiplicity of infection (MOI) of 100 for 2 hours at 37°C under 5% CO_2_ and 95% humidity. The bacterial suspension was then removed and epithelial cell monolayers were rinsed 3 times with PBS to remove non-adherent bacteria. Next, the epithelial cells were fixed with PFA 4%. All bacterial cells remaining adherent to the surface of the epithelial cells were stained for 1 h with a rabbit anti-*L*. *monocytogenes* polyclonal antibody (Meridian Life Science^®^, Memphis, USA) counterstained with a secondary antibody goat anti-rabbit conjugated with AlexaFluor 568 (Invitrogen, Life Technology^®^, Saint Aubin, France) for 30 min. After rinsing with PBS, the epithelial cells were permeabilized in 0.5% Triton X-100 in PBS for 10 min. All adherent and invading bacteria were stained with the same above-mentioned procedure but using an AlexaFluor 488 conjugated secondary antibody (Invitrogen, Life Technology^®^, Saint Aubin, France). The coverslips were then inverted and mounted on glass slides and were examined using a BX51 epifluorescence microscope (Olympus^®^, Tokyo, Japan). The percentages of adherent or invading bacteria were determined as follows: % adherent bacteria = Total number of stained bacteria (adherent + internalized, *i*.*e*. stained in green)/Total number of bacteria inoculated into Caco-2 cells; % invading bacteria = Number of invading bacteria/Total number of bacteria inoculated into Caco-2 cells; Number of invading bacteria = Total number of stained bacteria (adherent + internalized, *i*.*e*. stained in green)—Number of adherent bacteria (*i*.*e*. stained in red). 1. Olier M, Pierre F, Rousseaux S, Lemaitre JP, Rousset A, Piveteau P, et al. Expression of truncated Internalin A is involved in impaired internalization of some *Listeria monocytogenes* isolates carried asymptomatically by humans. Infect Immun 2003 Mar;71(3):1217–24.
(TIF)
