# Supplementary figures and images for: Intestinal Cell Tight Junctions Limit Invasion of Candida albicans through Active Penetration and Endocytosis in the Early Stages of the Interaction of the Fungus with the Intestinal Barrier
Source: PLoS One. 2016 Mar 2;11(3):e0149159. doi: 10.1371/journal.pone.0149159 (PMC4775037; doi:10.1371/journal.pone.0149159)

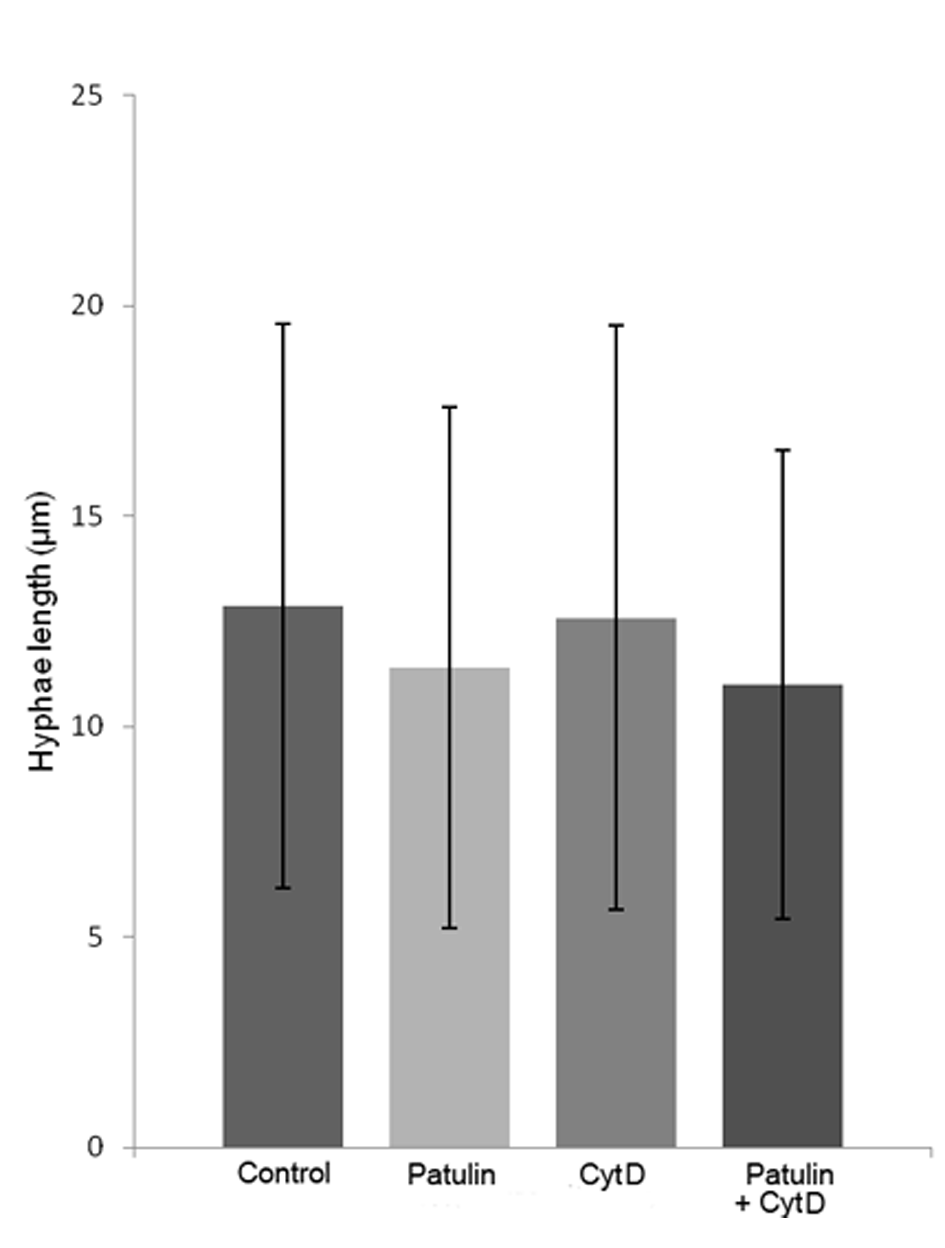

Supplement: S1 Fig — Filamentation assay were conducted with C. albicans SC5314 incubated in serum for 4 hours at 37°C in the presence of 100 μM patulin, 0.5μM cytochalasin or both and compared with yeasts incubated in the same conditions without any drugs. No effect of the drugs was observed on the number and the length of the hyphae formed (Statistics: Anova test). (TIF) [file pone.0149159.s001.tif]

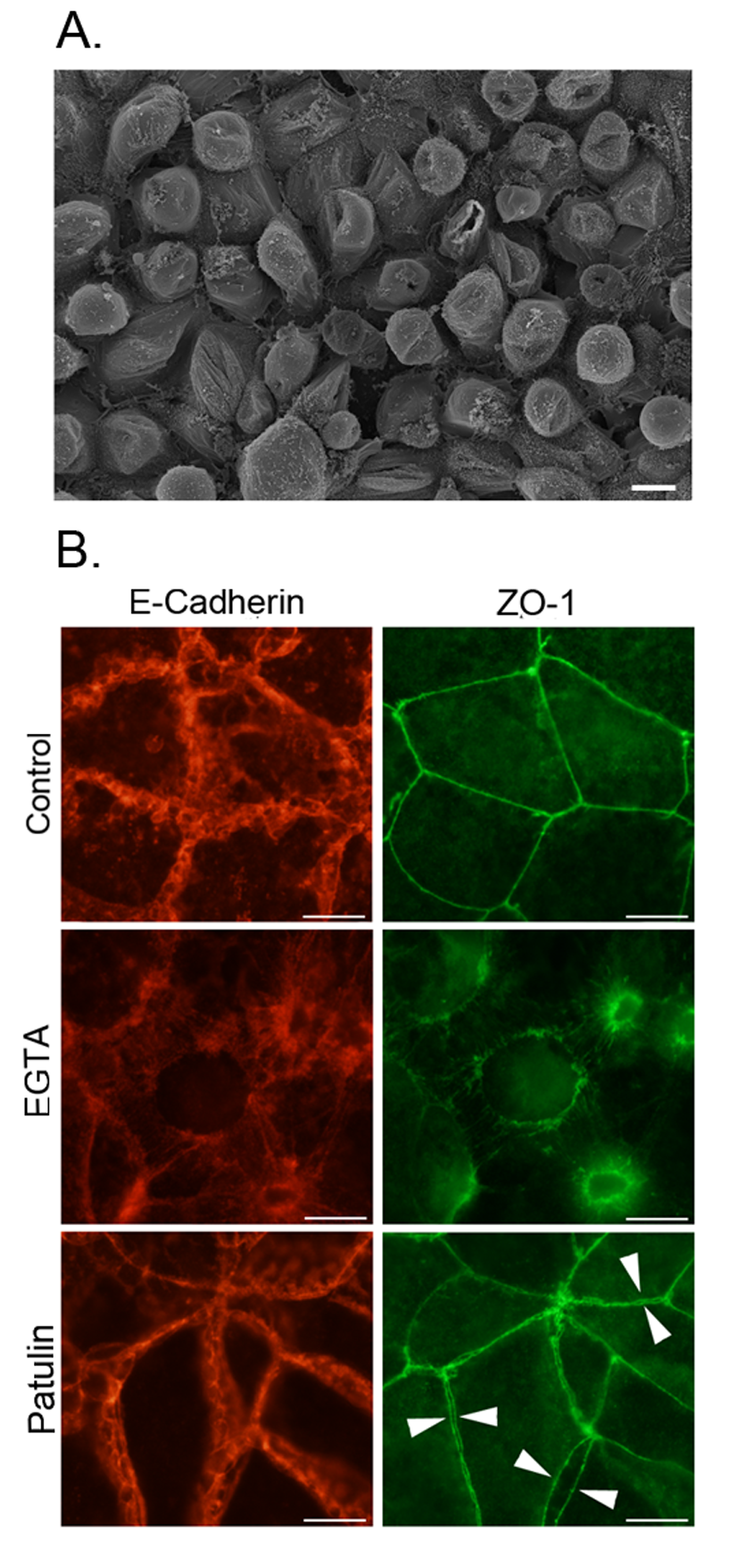

Supplement: S2 Fig — Differentiated Caco-2 cells were pre-treated with 2.5mM EGTA (Sigma-Aldrich®, Saint-Quentin Fallavier, France), a calcium chelator, in DMEM without calcium for 30 min at 37°C, 5% CO2. The medium was then replaced with fresh DMEM without calcium in order to maintain TJ in an open conformation. A. Fully released cellular junctions were observed in Caco-2 cells treated with 2.5 mM EGTA (Scale bar: 10 μm). B. Treatment with patulin specifically altered TJ integrity, whereas EGTA induced complete disruption of the cellular junctions (i.e. Adherens junctions) illustrated by the relocation of staining of both E-cadherin and ZO-1 (Scale bar: 10 μm). (TIF) [file pone.0149159.s002.tif]

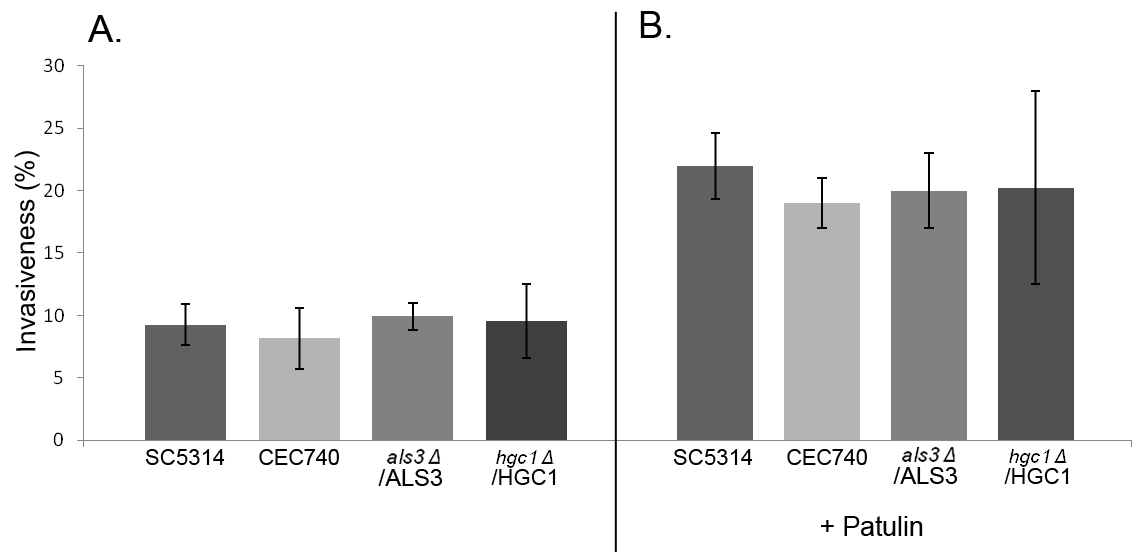

Supplement: S3 Fig — Caco-2 cells were infected with C. albicans for 2 hours after which the number of Candida cells partially internalized into enterocytes was determined using a differential fluorescence assay as described in the experimental procedures section. Results show the mean ± standard deviation of at least three independent experiments for each of which, 300 C. albicans’ interacting cells were checked for adherence to and/or invasion into Caco-2 cells (Statistics: Kruskal and Wallis’ test). (TIF) [file pone.0149159.s003.tif]

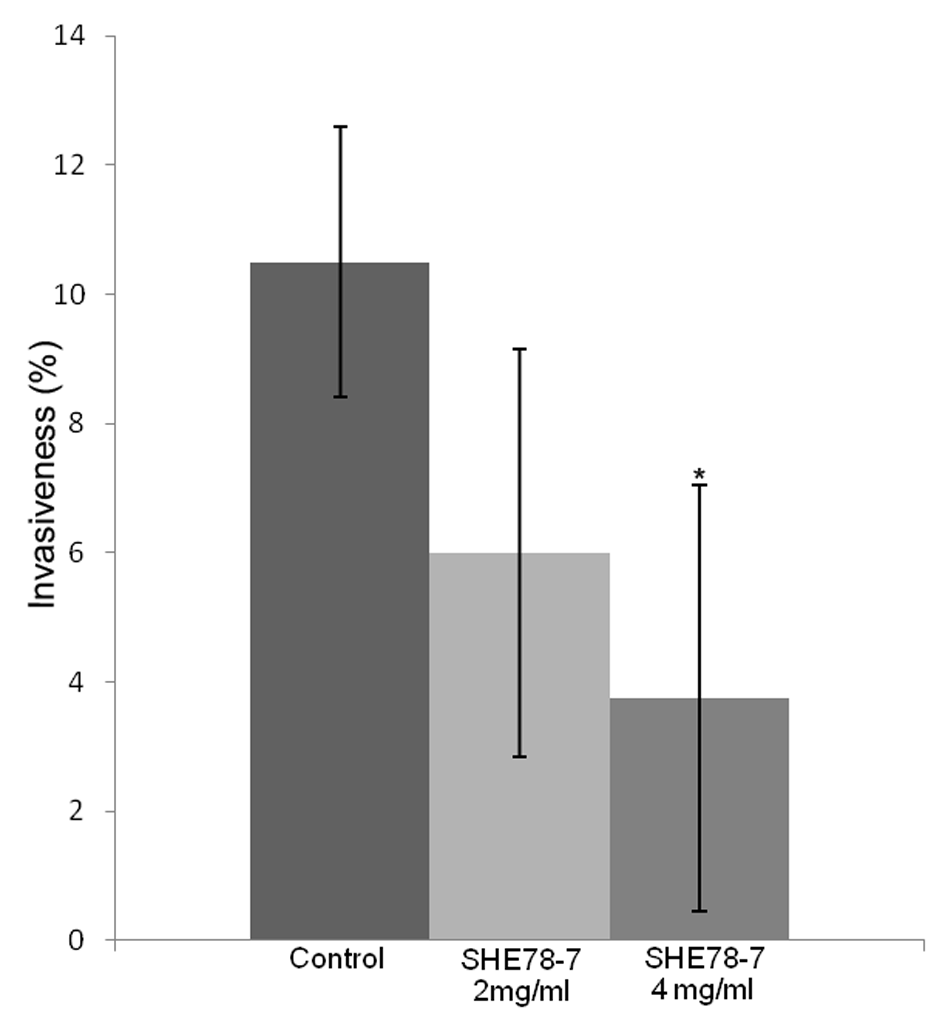

Supplement: S4 Fig — Differentiated Caco-2 enterocytes were inoculated with Listeria monocytogenes strain ScottA at a MOI of 100 in the presence of 2 and 4 mg/ml of the blocking antibody anti-E-cadherin SHE78-7. Significant differences were observed between the various conditions (*p<0.05, Anova test). Bacterial growth conditions and bacterial invasion assays: All experiments performed with Listeria monocytogenes (L. monocytogenes) were carried out with L. monocytogenes strain ScottA (Institut Pasteur Collection, Paris, France) [1]. Bacteria were grown routinely on blood agar plates. For infection experiments, bacteria were grown overnight in Brain Heart Infusion (BHI) at 37°C. L. monocytogenes cells were then diluted in fresh BHI and grown for 2 to 3 hours at 37°C to obtain an optical density between 0.20 and 0.30 at 600 nm. Bacterial suspensions were then adjusted to the desired concentration in DMEM. Adhesion and invasion assays were performed using a multiplicity of infection (MOI) of 100 for 2 hours at 37°C under 5% CO2 and 95% humidity. The bacterial suspension was then removed and epithelial cell monolayers were rinsed 3 times with PBS to remove non-adherent bacteria. Next, the epithelial cells were fixed with PFA 4%. All bacterial cells remaining adherent to the surface of the epithelial cells were stained for 1 h with a rabbit anti-L. monocytogenes polyclonal antibody (Meridian Life Science®, Memphis, USA) counterstained with a secondary antibody goat anti-rabbit conjugated with AlexaFluor 568 (Invitrogen, Life Technology®, Saint Aubin, France) for 30 min. After rinsing with PBS, the epithelial cells were permeabilized in 0.5% Triton X-100 in PBS for 10 min. All adherent and invading bacteria were stained with the same above-mentioned procedure but using an AlexaFluor 488 conjugated secondary antibody (Invitrogen, Life Technology®, Saint Aubin, France). The coverslips were then inverted and mounted on glass slides and were examined using a BX51 epifluorescence microscope (Olymp [file pone.0149159.s004.tif]
